# Neonatal Thrombocytopenia after Perinatal Asphyxia Treated with Hypothermia: A Retrospective Case Control Study

**DOI:** 10.1155/2014/760654

**Published:** 2014-08-21

**Authors:** N. Boutaybi, F. Razenberg, V. E. H. J. Smits-Wintjens, E. W. van Zwet, M. Rijken, S. J. Steggerda, E. Lopriore

**Affiliations:** ^1^Division of Neonatology, Department of Pediatrics, Leiden University Medical Center, J6-S, Albinusdreef 2, 2333 ZA Leiden, The Netherlands; ^2^Department of Medical Statistics, Leiden University Medical Center, Albinusdreef 2, 2333 ZA Leiden, The Netherlands

## Abstract

Our objective was to estimate the effect of therapeutic hypothermia on platelet count in neonates after perinatal asphyxia. We performed a retrospective case control study of all (near-) term neonates with perinatal asphyxia admitted between 2004 and 2012 to our neonatal intensive care unit. All neonates treated with therapeutic hypothermia were included in this study (hypothermia group) and compared with a historic control group of neonates with perinatal asphyxia treated before introduction of therapeutic hypothermia (2008). Primary outcome was thrombocytopenia during the first week after birth. Thrombocytopenia was found significantly more often in the hypothermia group than in the control group, 80% (43/54) versus 59% (27/46) (*P* = .02). The lowest mean platelet count in the hypothermia group and control group was 97 × 10^9^/L and 125 × 10^9^/L (*P* = .06), respectively, and was reached at a mean age of 4.1 days in the hypothermia group and 2.9 days in the control group (*P* < .001). The incidence of moderate/severe cerebral hemorrhage was 6% (3/47) in the hypothermia group versus 9% (3/35) in the control group (*P* = .64). In conclusion, neonates with perinatal asphyxia treated with therapeutic hypothermia are at increased risk of thrombocytopenia, without increased risk of cerebral hemorrhage.

## 1. Introduction

Thrombocytopenia, defined as a platelet count below 150 × 10^9^/L, occurs in 1 to 5% of healthy term neonates [1, 2]. The prevalence of thrombocytopenia is reported to be much higher in sick neonates, ranging from 22 to 35% in those admitted to neonatal intensive care units [1–4]. Thrombocytopenia is associated with an increased risk of pulmonary, gastrointestinal, and intraventricular hemorrhage (IVH) [5].

One of the most common causes of early-onset thrombocytopenia (<72 h of birth) in term neonates is perinatal asphyxia [2]. Perinatal asphyxia remains an important cause of morbidity and mortality of the full-term newborn and is still a major cause of death worldwide [6–10]. Therapeutic hypothermia, nowadays considered as the gold standard of treatment for perinatal asphyxia, reduces the risk of permanent brain injury and is associated with decreased rates of mortality and neurodevelopmental disability [6, 9, 11–13]. The protective effects of hypothermia are attributed primarily to a reduction of apoptosis and of inflammation [12].

In a recent Cochrane meta-analysis, therapeutic hypothermia was reported to increase the relative risk (RR) of thrombocytopenia in neonates with perinatal asphyxia (relative risk (RR): 1.21 (95% confidence interval (CI) 1.05–1.40)) compared to a control group [14]. However, research on the severity, course, and consequences of thrombocytopenia after therapeutic hypothermia is limited.

The aim of this study was to estimate the incidence, timing, and severity of thrombocytopenia during the first week after birth in neonates with perinatal asphyxia and to study the effect of therapeutic hypothermia on platelet count.

## 2. Methods

All (near-) term neonates (≥36 weeks' gestation) with perinatal asphyxia admitted to the tertiary neonatal intensive care unit of the Leiden University Medical Center from January 2004 to November 2012 were eligible for the study. In the hypothermia group we included all neonates with perinatal asphyxia treated with therapeutic hypothermia. In our neonatal intensive care unit treatment with hypothermia was introduced as standard management for perinatal asphyxia in September 2008. Neonates were cooled by whole-body hypothermia at a degree of 33.5°C for 72 hours. They were nursed on a cooling blanket in which fluid was circulating; the temperature of the fluid was regulated automatically (Criticool, MTRE Advanced technologies). After 72 hours we rewarmed them in intervals of 0.3°C per 60 minutes till a body temperature of 36.5°C was reached. The selection criteria for therapeutic hypothermia used in our center are shown in Box 1. Neonates with perinatal asphyxia admitted to our neonatal intensive care unit before October 2008 were not treated with therapeutic hypothermia and were included in the control group.

The primary aim of the study was to compare the incidence, severity, and course of thrombocytopenia between the hypothermia and the control group. Thrombocytopenia was defined as a platelet count below 150 × 10^9^/L. The severity of thrombocytopenia was classified in four categories, based on the lowest platelet count in the first week after birth: mild (platelet count 100–149 × 10^9^/L), moderate (platelet count 50–99 × 10^9^/L), severe (platelet count 30–49 × 10^9^/L), and very severe (platelet count < 30 × 10^9^/L). Data were extracted from our patient database and laboratory files. We recorded neonatal, laboratory, neurologic morbidity, and hematologic data in both groups.

### 2.1. Neonatal and Laboratory Data

The following neonatal and laboratory data were recorded: birth weight, gestational age at birth, gender, mode of delivery, Apgar score at 5 minutes, intubation in the delivery room, blood gas pH, base excess, and lactate level. Measurements of the laboratory data were performed either in arterial umbilical cord blood or in arterial or capillary blood gas within one hour after birth.

### 2.2. Hematologic Data

The following hematologic laboratory data were recorded: platelet count (at day 1 till day 7 after birth), prothrombin time (PT), activated partial thromboplastin time (APTT), international normalized ratio (INR), and fibrinogen level. As for hematologic treatment we recorded treatment with fresh frozen plasma (FFP), red blood cell transfusions, and/or platelet transfusions. In our neonatal intensive care unit, platelet transfusions are given when: (1) platelet count is below 30 × 10^9^/L, stable infant, (2) platelet count is below 50 × 10^9^/L, unstable infant, previous major bleeding or before planned surgery, or (3) platelet count is below 100 × 10^9^/L in neonates with active bleeding [4].

### 2.3. Mortality, Neurologic Morbidity, and Other Clinical Data

The following postnatal data were recorded: mortality within 1 month after birth, neonatal seizures, treatment with anticonvulsants, neonatal sepsis (defined as a positive blood culture in a neonate with clinical signs of infection), small for gestational age (SGA, defined as a birth weight < 10th centile), days on mechanical ventilation, number of hospital days in our neonatal intensive care unit, and hemorrhage on cranial ultrasound and/or magnetic resonance imaging (MRI). Cranial ultrasound scan (CUS) results were assessed for IVH and parenchymal hemorrhage. IVH was classified according to Papile et al. [15]. MRI scans were reviewed for presence of subdural, intraparenchymal, and/or intraventricular hemorrhage.

We classified mild hemorrhage as mild IVH (grade 1), punctate parenchymal hemorrhage, and minor subdural bleeds without parenchymal compression and/or shift detected by CUS and/or MRI. Moderate/severe IVH (≥grade 2), larger parenchymal hemorrhage, and large subdural hemorrhage causing parenchymal compression and/or shift were classified as moderate/severe hemorrhage.

### 2.4. Statistical Analysis

We calculated that group sizes of at least 45 infants were required to demonstrate a 25% difference in incidence of thrombocytopenia (75% versus 50%) with a significance of 0.05 and a power of 80%, by one-tailed analysis. Chi-square tests or Fisher's exact tests were applied to analyze categorical variables, as appropriate. For comparison of continuous variables, independent-sample *t*-test was used. Odds ratios (OR) and 95% CI were calculated by univariate logistic regression. All reported *P* values were two-sided and were considered statistically significant at *P* < .05. Data analyses were performed using SPSS Statistics software (version 20.0, SPSS Inc., Chicago, IL, USA).

## 3. Results

**Figure 1:**
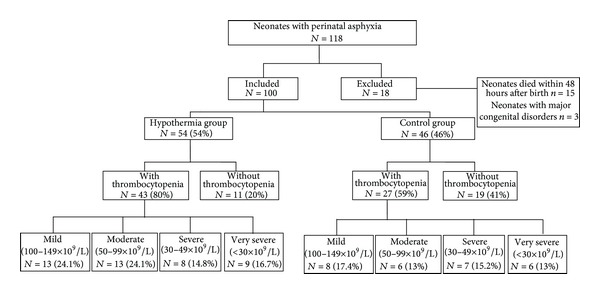
Flowchart showing the derivation of our population and the severity of thrombocytopenia.

During the study period, 118 (near-) term neonates (≥36 weeks' gestation) with perinatal asphyxia were admitted to our neonatal intensive care unit. Neonates who died within 48 hours after birth (*n* = 15) and neonates with major congenital disorders (*n* = 3) were excluded. A total of 100 neonates met our inclusion criteria. In the hypothermia group 54 neonates were included and 46 neonates, born in the period before introduction of therapeutic hypothermia, were included in the control group. The criteria for perinatal asphyxia were the same in the two groups. Baseline characteristics were similar between the hypothermia group and the control group, except for lower Apgar scores in neonates in the hypothermia group (see Table 1). The flow chart showing the derivation of our population and the severity of thrombocytopenia is shown in Figure 1.

**Figure 2:**
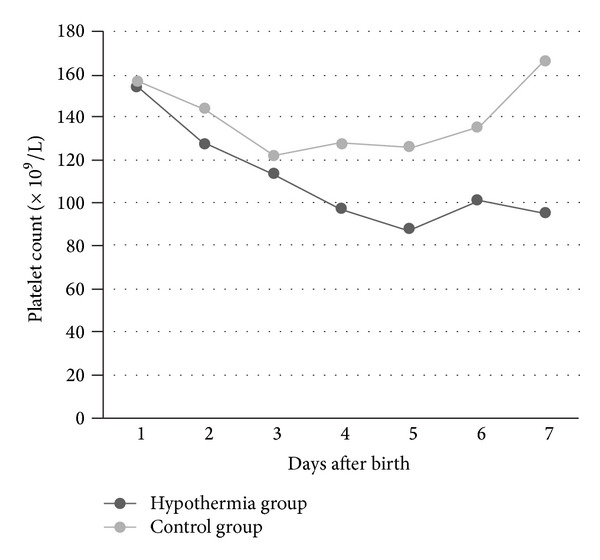
Mean platelet count during the first week after birth in our study population.

Platelet counts were measured in 74/100 (74%) neonates at day 1 (day of birth) and at least once in the first week after birth in all neonates (100%). At day 1, the mean platelet count in the hypothermia group was 154 × 10^9^/L compared to 156 × 10^9^/L in the control group (*P* = .90). The lowest platelet count during the first week after birth was in the hypothermia group 97 × 10^9^/L compared to 125 × 10^9^/L in the control group (*P* = .06). The incidence of thrombocytopenia (<150 × 10^9^/L) was significantly higher in the hypothermia group than in the control group, 80% (43/54) versus 59% (27/46), respectively (OR 2.75, 95% CI 1.14–6.66, *P* = .02). The subdivision of thrombocytopenia in mild, moderate, severe, and very severe was similar between the hypothermia group and the control group: 24% (13/54) versus 17% (8/46) (*P* = .41), 24% (13/54) versus 13% (6/46) (*P* = .16), 15% (8/54) versus 15% (7/46) (*P* = .96), and 17% (9/54) versus 13% (6/46) (*P* = .61), respectively. The lowest platelet count in the hypothermia group was reached later compared to in the control group: at a mean age of 4.1 days (at day 5) versus 2.9 days (at day 3), after birth (*P* < .001) (see Figure 2). Coagulation data were collected from both groups. No significant differences in mean values of PT, APTT, INR, and fibrinogen were found between both groups. Further details on hematologic outcome in the study population are shown in Table 2.

### 3.1. Mortality, Neurologic Morbidity, and Other Clinical Data

An overview of neurologic morbidity, clinical findings, and presence of hemorrhage on neuroimaging in both groups is presented in Table 3. Mortality rate in the hypothermia group was 31% (17/54) compared to 20% (9/46) in the control group (OR 1.89, 95% CI 0.75–4.78, *P* = .18). In the hypothermia group 83% (45/54) of the neonates were treated with anticonvulsants, compared to 74% (34/46) in the control group, with phenobarbital being the most commonly administered drug (alone or in combination with other drugs). The number of neonates requiring more than 2 types of anticonvulsants, number of hospital days, and number of ventilation days were similar in both groups. CUS was performed in all neonates. The incidence of IVH grades II-III was similar in both groups, 2% (1/54) in the hypothermia group and 2% (1/46) in the control group (OR 0.87, 95% CI 0.05–14.24, *P* = .92). On CUS no subdural and intraparenchymal bleedings were detected. MRI was performed in 82% (82/100) of neonates (hypothermia group 47/54; control group 35/46). The overall incidence of intracranial hemorrhage detected on MRI was similar in both groups, 23% (11/47) in the hypothermia group versus 23% (8/35) in the control group (OR 1.03, 95% CI 0.37–2.91, *P* = .95) and hemorrhages were mostly mild (Table 3). The incidence of moderate/severe hemorrhage was similar in both groups, 6% (3/47) versus 9% (3/35) (*P* = .64), respectively.

## 4. Discussion

This study shows that the vast majority (80%) of neonates with perinatal asphyxia treated with hypothermia develop thrombocytopenia. This is an important topic especially since there is a movement to cool babies of lower gestational age without adequate safety data. The incidence of thrombocytopenia was almost threefold higher (OR 2.75) than in the control group with perinatal asphyxia treated without therapeutic hypothermia. The higher incidence of thrombocytopenia in the hypothermic group appears to be primarily an increase in the numbers of infants with mild or moderate thrombocytopenia.

Despite the increased rate of thrombocytopenia in the hypothermia group, we found no increased risk of intracranial hemorrhage. This is in accordance with previous studies showing no association between thrombocytopenia and the occurrence of major intracranial hemorrhage [4, 5, 16].

The incidence of thrombocytopenia in neonates treated with therapeutic hypothermia reported in the literature varies greatly from 3% to 65% [6–10, 17]. Differences in reported incidence are probably related to methodological differences between the studies, including different definitions for perinatal asphyxia, differences in timing of platelet count measurement, and variations in number of included patients. We found a difference in the course of platelet count between the hypothermia group and control group (see Figure 2). The lowest platelet count in the hypothermia group was reached at a later stage compared to the control group (day 5 versus day 3). This difference suggests an additional effect of hypothermia on platelet count in infants with perinatal asphyxia.

Our findings are in agreement with recent studies reported in the literature. In a recent Cochrane review (2013), eight randomized studies comparing platelet count in the hypothermia group with a control group were identified [14]. Meta-analysis of these eight trials showed a relative risk of thrombocytopenia in the hypothermia group of 1.21 (95% CI 1.05–1.40) compared to the control group [14]. This was in accordance with another systematic review from Shah et al. in 2007 (RR 1.51, 95% CI 1.09–2.10) [12]. In this review thrombocytopenia was defined as a platelet count below 100 × 10^9^/L and three randomized studies were analyzed [7, 8, 17]. Up to now, no individual randomized study found a difference in thrombocytopenia in the hypothermia group as compared to the control group [6–10, 17]. This correlation was only found in reviews after meta-analysis of different randomized studies [12, 14].

According to the recent Cochrane review, the mode of hypothermia (head cooling with mild systemic versus whole-body) may influence the incidence of thrombocytopenia. A slight increase (statistical) in risk of thrombocytopenia was detected in the group of infants treated with selective head cooling [14].

Hypothermia is known to decrease platelet function and platelet number [18]. The production of thromboxane B2, which has a role in clot formation, is dependent on temperature: lowering of the body temperature causes a reversible platelet dysfunction [18]. Hypothermia is also known to increase PT and APTT [19]. Enzymatic reactions of the coagulation cascade are inhibited by hypothermia [19]. However, in our study we found no differences in PT, APTT, and number of neonates requiring FFP transfusions. The occurrence of coagulation disorders was similar in both groups.

Because of the retrospective design the results of this study should be interpreted with care. Despite the fact that both groups in our study had the same criteria for perinatal asphyxia, the hypothermia group had lower Apgar scores. The higher incidence of thrombocytopenia in the hypothermia group could partly be due to the more severe degree of perinatal asphyxia. However, arterial cord blood values and other clinical parameters were similar in both groups suggesting that differences between groups were probably minimal. A further limitation of our study is the small number (*n* = 100) of included neonates. Larger prospective studies should be conducted to determine the exact mechanism that contributes to the occurrence of thrombocytopenia by hypothermia. Randomized controlled trials are not ethical anymore because of the proven protective effect of therapeutic hypothermia.

In conclusion, this is the first study focusing on the incidence, severity, and course of thrombocytopenia after treatment with hypothermia. We conclude that therapeutic hypothermia increases the risk of thrombocytopenia in neonates after perinatal asphyxia, without increased risk of cerebral hemorrhage. Thrombocytopenia lasts longer and the nadir of platelet count is reached a couple of days later in neonates treated with hypothermia.

## Figures and Tables

**Box 1:**
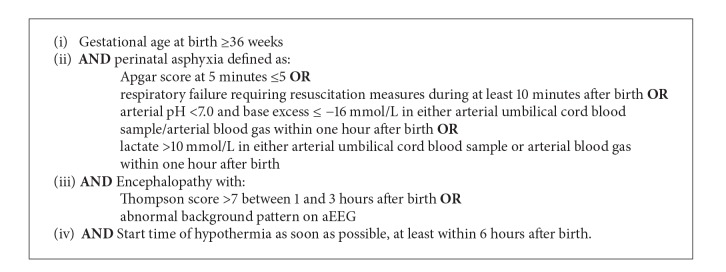
Selection criteria for therapeutic hypothermia at the Leiden University Medical Center.

**Table 1:** Baseline characteristics of the study population.

	Hypothermia group	Control group	*P* value
	*n* = 54	*n* = 46	
Birth weight—grams∗	3446 (625)	3352 (606)	.45
Gestational age—weeks∗	39.4 (1.55)	39.8 (1.85)	.28
Male gender—*n* (%)	27 (50%)	18 (39%)	.28
Caesarean delivery—*n* (%)	27 (50%)	28 (61%)	.28
Vacuum extraction—*n* (%)	16 (30%)	15 (33%)	.75
Forceps delivery—*n* (%)	2 (4%)	3 (7%)	.66
Shoulder dystocia—*n* (%)	4 (7%)	1 (2%)	.37
Apgar at 5 min ≤ 5—*n* (%)	51 (96%)	31 (67%)	.00
Arterial cord blood/blood gas			
pH <7.0—*n* (%)	39 (72%)	34 (74%)	.85
Base excess ≤−16 mmol/L—*n* (%)	33 (61%)	24 (52%)	.37
Lactate >10 mmol/L—*n* (%)	33 (61%)	22 (48%)	.18

∗Value given as mean (SD).

**Table 2:** Hematologic results of the study population.

	Hypothermia	Control group	*P* value	OR [95% CI]
	group *n* = 54	*n* = 46		
Platelet count/transfusions				
Platelet count <150 × 10^9^/L∗	43 (80%)	27 (59%)	.02	2.75 [1.14–6.66]
Platelet count at birth—×10^9^/L∗	154 (77)	157 (81)	.90	1.00 [0.99–1.01]
Lowest platelet count in the first week after birth∗	97 (62)	125 (78)	.06	0.99 [0.99–1.00]
Day lowest platelet count∗	4.1 (1.8)	2.9 (1.4)	<.001	1.58 [1.21–2.06]
Neonates requiring platelet transfusions—*n* (%)	15 (28%)	10 (22%)	.49	1.39 [0.55–3.47]
Number. of platelet transfusions per	0.00 (1)	0.00 (0)	.94	0.99 [0.74–1.32]
neonate∗∗				
Coagulation disorders/FFP transfusions				
PT—seconds∗	25.3 (15.0)	21.2 (16.7)	.27	1.02 [0.98–1.06]
INR∗	1.8 (1.1)	1.8 (1.5)	.96	0.99 [0.69–1.43]
APTT—seconds∗	50.0 (20.1)	42.6 (23.8)	.16	1.02 [0.99–1.04]
Fibrinogen—gram/L∗	1.6 (0.8)	2.0 (0.9)	.08	0.60 [0.34–1.07]
Neonates requiring FFP	13 (24%)	8 (17%)	.41	1.51 [0.56–4.03]
transfusions—*n* (%)				
Number of FFP transfusions per	0.00 (0)	0.00 (0)	.81	0.95 [0.62–1.45]
neonate∗∗				

PT: prothrombin time; INR: international normalized ratio; APTT: activated partial thromboplastin time; FFP: fresh frozen plasma.

∗∗Value given as median (IQR).

**Table 3:** Morbidity, clinical findings, and presence of hemorrhage on neuroimaging.

	Hypothermia	Control	*P* value	OR [95% CI]
	group *n* = 54	group *n* = 46		
Seizures—*n* (%)	45 (83%)	34 (74%)	.25	1.77 [0.67–4.67]
Treatment with >2 types of anticonvulsants—*n* (%)	18 (33%)	8 (18%)	.07	2.38 [0.92–6.14]
Neonatal sepsis	3 (6%)	2 (4%)	.78	1.29 [0.20–8.10]
SGA	0 (0%)	2 (4%)	.12	∞
IVH grades II-III on cranial US—*n* (%)	1 (2%)	1 (2%)	.92	0.87 [0.05–14.24]
Cerebral bleeding on MRI—*n* (%)	11 (23%)	8 (23%)	.95	1.03 [0.37–2.91]
Mild	8 (17%)	5 (14%)	.64	
Moderate/severe	3 (6%)	3 (9%)	.64	
Type of moderate/severe hemorrhage				
Subdural	0 (0%)	0 (0%)	—	
Intraparenchymal	2 (4%)	2 (6%)	.74	
Intraventricular	2 (4%)	1 (3%)	.76	
Mortality—*n* (%)	17 (31%)	9 (20%)	.18	1.89 [0.75–4.78]
Mechanical ventilation days∗	4.8 (2.94)	3.6 (3.40)	.07	1.13 [0.99–1.28]
Hospital days∗	10.7 (7.07)	10.4 (8.46)	.89	1.00 [0.95–1.06]

SGA: small for gestational age; IVH: intraventricular haemorrhage; US: ultrasound; MRI: magnetic resonance imaging; ∞: infinite (because of zero value in one group); ∗value given as mean (SD).

## Abbreviations

APTT: Activated partial thromboplastin time
CI: Confidence intervals
CUS: Cranial ultrasound scan
FFP: Fresh frozen plasma
INR: International normalized ratio
IVH: Intraventricular hemorrhage
MRI: Magnetic resonance imaging
OR: Odds ratios
PT: Prothrombin time
RR: Relative risk
SGA: Small for gestational age.
